# (3*S*,3a*S*,5a*S*,7*S*,8*S*,10a*S*,10b*R*)-7,8-Dihydr­oxy-3-isopropyl-5a,8-dimethyl-2,3,4,5,5a,6,7,8,10a,10b-deca­hydro­cyclo­hepta­[*e*]indene-3a(1*H*)-carboxylic acid

**DOI:** 10.1107/S1600536808018941

**Published:** 2008-06-28

**Authors:** Iván Brito, Jorge Bórquez, Luis Alberto Loyola, Alejandro Cárdenas, Matías López-Rodríguez

**Affiliations:** a Departamento de Química, Facultad de Ciencias Básicas, Universidad de Antofagasta, Casilla 170, Antofagasta, Chile; b Departamento de Física, Facultad de Ciencias Básicas, Universidad de Antofagasta, Casilla 170, Antofagasta, Chile; c Instituto de Bio-Orgánica ’Antonio González’, Universidad de La Laguna, Astrofísico Francisco Sánchez N°2, La Laguna, Tenerife, Spain

## Abstract

The mol­ecule of the title compound, C_20_H_32_O_4_, is built up from three fused five-membered, six-membered and seven-membered rings. The five-membered ring has an envelope conformation, whereas the six- and seven-membered rings have chair conformations. The crystal structure is stabilized by strong inter­molecular O—H⋯O hydrogen bonds, forming a three-dimensional network. The absolute configuration was assigned on the basis of earlier chemical studies.

## Related literature

For related literature, see: Araya *et al.* (2003 ▶); Cremer & Pople (1975 ▶); Fuentes *et al.* (2005 ▶); Loyola *et al.* (1996 ▶, 2004 ▶); Wickens (1995 ▶).
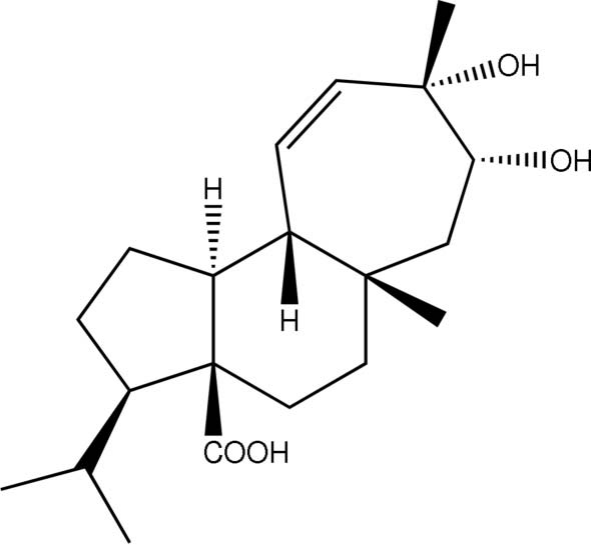


## Experimental

### 

#### Crystal data

C_20_H_32_O_4_
*M* *_r_* = 336.46
Orthorhombic, 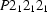

*a* = 11.094 (7) Å
*b* = 12.728 (10) Å
*c* = 13.8776 (11) Å
*V* = 1959.6 (19) Å^3^
*Z* = 4
Mo *K*α radiation
μ = 0.08 mm^−1^
*T* = 298 (2) K
0.30 × 0.20 × 0.10 mm

#### Data collection

Nonius KappaCCD area-detector diffractometer
Absorption correction: none
9149 measured reflections
1922 independent reflections
1836 reflections with *I* > 2σ(*I*)
*R*_int_ = 0.072

#### Refinement

*R*[*F* ^2^ > 2σ(*F* ^2^)] = 0.039
*wR*(*F* ^2^) = 0.117
*S* = 1.13
1922 reflections
226 parameters
H-atom parameters constrained
Δρ_max_ = 0.21 e Å^−3^
Δρ_min_ = −0.14 e Å^−3^

### 

Data collection: *COLLECT* (Nonius, 2000 ▶); cell refinement: *DENZO-SMN* (Otwinowski & Minor, 1997 ▶); data reduction: *DENZO-SMN*; program(s) used to solve structure: *SIR97* (Altomare *et al.*, 1999 ▶); program(s) used to refine structure: *SHELXL97* (Sheldrick, 2008 ▶); molecular graphics: *ORTEP-3 for Windows* (Farrugia, 1997 ▶) and *PLATON* (Spek, 2003 ▶); software used to prepare material for publication: *WinGX* (Farrugia, 1999 ▶).

## Supplementary Material

Crystal structure: contains datablocks global, I. DOI: 10.1107/S1600536808018941/bt2730sup1.cif

Structure factors: contains datablocks I. DOI: 10.1107/S1600536808018941/bt2730Isup2.hkl

Additional supplementary materials:  crystallographic information; 3D view; checkCIF report

## Figures and Tables

**Table 1:** Hydrogen-bond geometry (Å, °)

*D*—H⋯*A*	*D*—H	H⋯*A*	*D*⋯*A*	*D*—H⋯*A*
O1—H1⋯O4^i^	0.82	1.80	2.613 (3)	170
O3—H3⋯O2^ii^	0.82	2.01	2.825 (3)	173
O4—H4⋯O3^iii^	0.82	1.94	2.752 (3)	172
C1—H1*B*⋯O2	0.97	2.44	2.889 (4)	108
C5—H5*B*⋯O1	0.97	2.51	3.054 (4)	116
C6—H6*B*⋯O4	0.97	2.52	2.901 (3)	104
C16—H16*A*⋯O3	0.96	2.46	2.809 (4)	101

Symmetry codes: (i) 
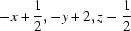
; (ii) 
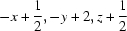
; (iii) 
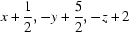
.

**Fig. 1.**
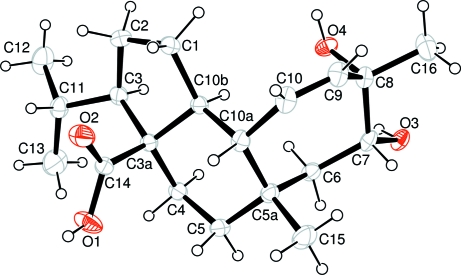
The molecule of compound (I), showing the atom-labelling scheme. Displacement ellipsoids are drawn at the 30% probability level and H atoms are shown as small spheres of arbitrary radii.

## supplementary crystallographic information

### Comment

Azorella compacta is a compact resinous cushion shrub that grows in the Andes of Peru, Bolivia, Argentina and Chile and has been used in folk medicine. The common name llareta is used for several species of the genus Azorella (Wickens, 1995). Mulinane diterpenes exhibits antiplasmodial (Loyola *et al.*, 2004), anti-Tripanosoma cruzi (Araya *et al.*, 2003) and antihyperglycemic (Fuentes *et al.*, 2005) activities.We have undertaken the X-ray crystal-structure determination of the title compound in order to establish its molecular conformation and relative stereochemistry. We are not able to determine the absolute stereochemistry by X-ray methods and the configuration shown here was chosen to be in accord with that reported in previous chemical studies (Loyola *et al.*, 1996). The structure consists of a mulinic acid skeleton and the isopropyl, methyl groups and carboxylic acid at C3, C5a, C8 and C3b are α-oriented respectively, whereas the hydroxyl groups at C8 and C7 are β-oriented. The cyclopentane (A), cyclohexane (B) and cycloheptene (C) rings are in an envelope, chair and chair conformation respectively [Q_2 _= 0.435 (2) Å, φ_2_= 118.7 (3)° for rig A; Q_T_= 0.581 (2) Å, θ = 174.4 (2)°, φ=131 (2)° for ring B; Q_T_= 0.634 (2) Å, φ_2_=78.4 (6)°, for ring C] (Cremer & Pople, 1975). The A and B and B and C rings are *trans* and *cis*-fused respectively. The molecular conformation is stabilized by four intramolecular hydrogen bonds and  the crystal structure   is stabilized by   three intermolecular hydrogen bonds (Table 1).

### Experimental

Dried and finely powdered whole plant of Azorella compacta (3,0 kg) were extracted with petroleum ether at room temperature. After filtration, the solvent was evaporated in vacuum yielding a gum (220 g). The concentrated petrol ether extract was adsorbed on silica gel (300 g) and slurried onto the top of a column containing silica gel (2.0 kg) in petroleum ether, and eluted with a petroleum ether/ethyl acetate gradient with increasing amounts of ethyl acetate to produce six fractions. Fraction 2 (100 g) eluted with petroleum ether/ethyl acetate(18:2) was further separated and purified by silica gel column chromatography(petroleum ether/ethyl acetate), 19:1) to give 600 mg of the title compound. The structure were elucidated by analysis of their spectroscopic data. Recrystallization from hexane-ethyl acetate (7:3) at room temperature afforded colourless crystals suitable for X-ray diffraction analysis.

### Refinement

All H atoms were located on a difference Fourier map and then treated as riding atoms, with C - H bond lengths in the range 0.96 - 0.98 Å and O - H distances of 0.82 Å. For methyl atoms, *U*_iso_(H) = 1.5*U*_eq_(C), while for other H atoms, *U*_iso_(H) = 1.2*U*_eq_(C, O).   In the absence of significant anomalous scattering effects, Friedel pairs were averaged. The absolute configuration shown here was chosen to be in accord with that reported in previous chemical studies (Loyola *et al.*, 1996).

### Crystal data

**Table:**

C_20_H_32_O_4_	*F*_000_ = 736
*M**_r_* = 336.46	*D*_x_ = 1.14 Mg m^−^^3^
Orthorhombic, *P*2_1_2_1_2_1_	Mo *K*α radiation λ = 0.71073 Å
Hall symbol: P 2ac 2ab	Cell parameters from 5686 reflections
*a* = 11.094 (7) Å	θ = 2.3–25.2º
*b* = 12.728 (10) Å	µ = 0.08 mm^−^^1^
*c* = 13.8776 (11) Å	*T* = 298 (2) K
*V* = 1959.6 (19) Å^3^	Block, colorless
*Z* = 4	0.30 × 0.20 × 0.10 mm

### Data collection

**Table:**

Nonius KappaCCD area-detector diffractometer	1836 reflections with *I* > 2σ(*I*)
Radiation source: fine-focus sealed tube	*R*_int_ = 0.072
Monochromator: graphite	θ_max_ = 25.2º
φ scans, and ω scans with κ offsets	θ_min_ = 2.4º
Absorption correction: none	*h* = −9→13
9149 measured reflections	*k* = −11→15
1922 independent reflections	*l* = −15→16

### Refinement

**Table:**

Refinement on *F*^2^	H-atom parameters constrained
Least-squares matrix: full	*w* = 1/[σ^2^(*F*_o_^2^) + (0.0775*P*)^2^ + 0.1473*P*] where *P* = (*F*_o_^2^ + 2*F*_c_^2^)/3
*R*[*F*^2^ > 2σ(*F*^2^)] = 0.039	(Δ/σ)_max_ = 0.005
*wR*(*F*^2^) = 0.117	Δρ_max_ = 0.21 e Å^−^^3^
*S* = 1.13	Δρ_min_ = −0.14 e Å^−^^3^
1922 reflections	Extinction correction: none
226 parameters	

### Special details

**Table:**

Geometry. All e.s.d.'s (except the e.s.d. in the dihedral angle between two l.s. planes) are estimated using the full covariance matrix. The cell e.s.d.'s are taken into account individually in the estimation of e.s.d.'s in distances, angles and torsion angles; correlations between e.s.d.'s in cell parameters are only used when they are defined by crystal symmetry. An approximate (isotropic) treatment of cell e.s.d.'s is used for estimating e.s.d.'s involving l.s. planes.
Refinement. Refinement of *F*^2^ against ALL reflections. The weighted *R*-factor *wR* and goodness of fit *S* are based on *F*^2^, conventional *R*-factors *R* are based on *F*, with *F* set to zero for negative *F*^2^. The threshold expression of *F*^2^ > σ(*F*^2^) is used only for calculating *R*-factors(gt) *etc*. and is not relevant to the choice of reflections for refinement. *R*-factors based on *F*^2^ are statistically about twice as large as those based on *F*, and *R*- factors based on ALL data will be even larger.

### Fractional atomic coordinates and isotropic or equivalent isotropic displacement parameters (Å^2^)

**Table:**

	*x*	*y*	*z*	*U*_iso_*/*U*_eq_	
O1	0.22790 (18)	0.80481 (16)	0.67764 (12)	0.0550 (5)	
H1	0.2371	0.7969	0.6195	0.066 (2)*	
O2	0.39658 (15)	0.89409 (15)	0.66282 (11)	0.0497 (5)	
O3	0.01429 (13)	1.24195 (13)	1.01859 (10)	0.0361 (4)	
H3	0.0408	1.1986	1.0568	0.066 (2)*	
O4	0.27135 (13)	1.21546 (12)	0.99028 (10)	0.0352 (4)	
H4	0.3422	1.2321	0.9835	0.066 (2)*	
C1	0.44994 (19)	1.03680 (17)	0.81893 (16)	0.0368 (5)	
H1A	0.4778	1.0971	0.8554	0.046 (2)*	
H1B	0.4745	1.0446	0.7522	0.046 (2)*	
C2	0.4986 (2)	0.93369 (19)	0.86208 (19)	0.0431 (6)	
H2A	0.5396	0.9477	0.9224	0.046 (2)*	
H2B	0.5554	0.9014	0.818	0.046 (2)*	
C3	0.3898 (2)	0.85939 (16)	0.87930 (14)	0.0338 (5)	
H3A	0.3642	0.8699	0.9462	0.041*	
C3A	0.28837 (18)	0.90550 (15)	0.81408 (13)	0.0278 (4)	
C4	0.15886 (19)	0.88598 (16)	0.84672 (15)	0.0330 (4)	
H4A	0.1524	0.8994	0.9153	0.046 (2)*	
H4B	0.1381	0.813	0.8355	0.046 (2)*	
C5	0.0704 (2)	0.95643 (19)	0.79272 (17)	0.0407 (5)	
H5A	−0.0097	0.9439	0.8182	0.046 (2)*	
H5B	0.0698	0.9354	0.7255	0.046 (2)*	
C5A	0.09599 (19)	1.07500 (17)	0.79757 (15)	0.0343 (5)	
C6	0.06692 (18)	1.11212 (16)	0.90075 (14)	0.0329 (4)	
H6A	−0.0136	1.0875	0.9159	0.046 (2)*	
H6B	0.1217	1.0758	0.9438	0.046 (2)*	
C7	0.07182 (19)	1.22815 (17)	0.92647 (14)	0.0322 (4)	
H7	0.0232	1.2658	0.8787	0.039*	
C8	0.1969 (2)	1.27901 (16)	0.92731 (15)	0.0332 (5)	
C9	0.2535 (2)	1.28268 (17)	0.82860 (15)	0.0377 (5)	
H9	0.2827	1.3481	0.8099	0.045*	
C10	0.2678 (2)	1.20670 (18)	0.76493 (15)	0.0390 (5)	
H10	0.3086	1.2265	0.7093	0.047*	
C10A	0.22889 (19)	1.09300 (16)	0.76724 (13)	0.0319 (4)	
H10A	0.2354	1.0675	0.7008	0.038*	
C10B	0.31312 (18)	1.02422 (15)	0.82710 (13)	0.0286 (4)	
H10B	0.2936	1.0397	0.8945	0.034*	
C11	0.4236 (2)	0.74293 (17)	0.86931 (15)	0.0398 (5)	
H11	0.4606	0.7335	0.8058	0.048*	
C12	0.5173 (3)	0.7132 (3)	0.9447 (2)	0.0599 (8)	
H12A	0.5844	0.7607	0.9408	0.066 (2)*	
H12B	0.5448	0.6427	0.9331	0.066 (2)*	
H12C	0.482	0.7173	1.0077	0.066 (2)*	
C13	0.3176 (3)	0.6675 (2)	0.8759 (3)	0.0675 (9)	
H13A	0.2766	0.6777	0.9362	0.066 (2)*	
H13B	0.3463	0.5965	0.8721	0.066 (2)*	
H13C	0.2628	0.6808	0.8238	0.066 (2)*	
C14	0.31036 (18)	0.86941 (16)	0.71064 (13)	0.0301 (4)	
C15	0.0128 (3)	1.1305 (2)	0.72577 (19)	0.0545 (7)	
H15A	0.0256	1.1022	0.6625	0.066 (2)*	
H15B	0.0303	1.2044	0.7254	0.066 (2)*	
H15C	−0.0696	1.1198	0.7444	0.066 (2)*	
C16	0.1909 (3)	1.39039 (18)	0.9685 (2)	0.0525 (6)	
H16A	0.1586	1.388	1.0326	0.066 (2)*	
H16B	0.1399	1.433	0.9285	0.066 (2)*	
H16C	0.2704	1.42	0.9702	0.066 (2)*	

### Atomic displacement parameters (Å^2^)

**Table:**

	*U*^11^	*U*^22^	*U*^33^	*U*^12^	*U*^13^	*U*^23^
O1	0.0571 (10)	0.0727 (11)	0.0351 (9)	−0.0269 (10)	0.0080 (8)	−0.0216 (8)
O2	0.0426 (9)	0.0711 (12)	0.0353 (8)	−0.0131 (8)	0.0105 (7)	−0.0178 (8)
O3	0.0291 (7)	0.0475 (9)	0.0318 (8)	0.0110 (6)	0.0051 (6)	−0.0001 (6)
O4	0.0272 (7)	0.0449 (8)	0.0335 (7)	−0.0028 (7)	0.0012 (6)	0.0082 (6)
C1	0.0314 (11)	0.0364 (10)	0.0427 (11)	−0.0054 (9)	0.0010 (9)	−0.0081 (9)
C2	0.0300 (10)	0.0459 (12)	0.0534 (14)	0.0010 (10)	−0.0109 (10)	−0.0080 (10)
C3	0.0382 (11)	0.0376 (10)	0.0255 (9)	0.0046 (9)	−0.0058 (8)	−0.0040 (8)
C3A	0.0273 (9)	0.0316 (9)	0.0245 (9)	−0.0005 (8)	0.0013 (8)	−0.0027 (7)
C4	0.0304 (9)	0.0366 (10)	0.0320 (9)	−0.0052 (8)	0.0054 (8)	−0.0059 (8)
C5	0.0281 (10)	0.0482 (12)	0.0457 (12)	−0.0008 (9)	−0.0036 (9)	−0.0128 (10)
C5A	0.0308 (10)	0.0420 (11)	0.0302 (10)	0.0071 (9)	−0.0045 (8)	−0.0040 (8)
C6	0.0252 (9)	0.0400 (10)	0.0337 (10)	0.0016 (8)	0.0015 (8)	−0.0007 (8)
C7	0.0290 (9)	0.0411 (10)	0.0264 (9)	0.0112 (9)	0.0033 (8)	0.0017 (8)
C8	0.0369 (10)	0.0317 (9)	0.0310 (10)	0.0064 (9)	0.0034 (8)	0.0015 (8)
C9	0.0434 (12)	0.0321 (9)	0.0377 (11)	0.0021 (9)	0.0084 (9)	0.0100 (8)
C10	0.0462 (12)	0.0413 (11)	0.0294 (10)	0.0074 (10)	0.0113 (9)	0.0084 (8)
C10A	0.0372 (10)	0.0383 (10)	0.0201 (8)	0.0061 (9)	0.0026 (8)	−0.0012 (7)
C10B	0.0285 (10)	0.0310 (9)	0.0263 (9)	−0.0004 (8)	0.0019 (7)	−0.0029 (7)
C11	0.0444 (12)	0.0388 (11)	0.0362 (11)	0.0101 (10)	−0.0013 (10)	0.0028 (8)
C12	0.0706 (18)	0.0638 (16)	0.0454 (13)	0.0282 (15)	−0.0104 (13)	0.0082 (11)
C13	0.0611 (18)	0.0407 (13)	0.101 (2)	0.0003 (13)	0.0004 (17)	0.0144 (14)
C14	0.0301 (9)	0.0331 (9)	0.0272 (9)	0.0010 (8)	−0.0024 (8)	−0.0027 (7)
C15	0.0484 (13)	0.0693 (16)	0.0458 (13)	0.0188 (13)	−0.0178 (12)	−0.0044 (13)
C16	0.0619 (15)	0.0367 (12)	0.0588 (15)	−0.0006 (11)	0.0168 (13)	−0.0067 (11)

### Geometric parameters (Å, °)

**Table:**

O1—C14	1.312 (3)	C6—C7	1.520 (3)
O1—H1	0.82	C6—H6A	0.97
O2—C14	1.206 (3)	C6—H6B	0.97
O3—C7	1.440 (2)	C7—C8	1.531 (3)
O3—H3	0.82	C7—H7	0.98
O4—C8	1.449 (3)	C8—C9	1.508 (3)
O4—H4	0.82	C8—C16	1.530 (3)
C1—C10B	1.531 (3)	C9—C10	1.319 (3)
C1—C2	1.540 (3)	C9—H9	0.93
C1—H1A	0.97	C10—C10A	1.510 (3)
C1—H1B	0.97	C10—H10	0.93
C2—C3	1.551 (3)	C10A—C10B	1.526 (3)
C2—H2A	0.97	C10A—H10A	0.98
C2—H2B	0.97	C10B—H10B	0.98
C3—C11	1.535 (3)	C11—C12	1.523 (3)
C3—C3A	1.559 (3)	C11—C13	1.521 (4)
C3—H3A	0.98	C11—H11	0.98
C3A—C4	1.527 (3)	C12—H12A	0.96
C3A—C14	1.527 (3)	C12—H12B	0.96
C3A—C10B	1.546 (3)	C12—H12C	0.96
C4—C5	1.526 (3)	C13—H13A	0.96
C4—H4A	0.97	C13—H13B	0.96
C4—H4B	0.97	C13—H13C	0.96
C5—C5A	1.537 (3)	C15—H15A	0.96
C5—H5A	0.97	C15—H15B	0.96
C5—H5B	0.97	C15—H15C	0.96
C5A—C15	1.531 (3)	C16—H16A	0.96
C5A—C6	1.542 (3)	C16—H16B	0.96
C5A—C10A	1.550 (3)	C16—H16C	0.96
			
C14—O1—H1	109.5	O4—C8—C9	109.10 (16)
C7—O3—H3	109.5	O4—C8—C16	108.5 (2)
C8—O4—H4	109.5	C9—C8—C16	109.20 (18)
C10B—C1—C2	103.26 (18)	O4—C8—C7	106.57 (16)
C10B—C1—H1A	111.1	C9—C8—C7	112.58 (18)
C2—C1—H1A	111.1	C16—C8—C7	110.82 (19)
C10B—C1—H1B	111.1	C10—C9—C8	129.5 (2)
C2—C1—H1B	111.1	C10—C9—H9	115.3
H1A—C1—H1B	109.1	C8—C9—H9	115.3
C1—C2—C3	107.89 (18)	C9—C10—C10A	130.8 (2)
C1—C2—H2A	110.1	C9—C10—H10	114.6
C3—C2—H2A	110.1	C10A—C10—H10	114.6
C1—C2—H2B	110.1	C10—C10A—C10B	112.72 (18)
C3—C2—H2B	110.1	C10—C10A—C5A	114.76 (18)
H2A—C2—H2B	108.4	C10B—C10A—C5A	110.47 (16)
C11—C3—C2	112.66 (19)	C10—C10A—H10A	106.1
C11—C3—C3A	119.15 (17)	C10B—C10A—H10A	106.1
C2—C3—C3A	104.03 (17)	C5A—C10A—H10A	106.1
C11—C3—H3A	106.8	C10A—C10B—C1	120.45 (18)
C2—C3—H3A	106.8	C10A—C10B—C3A	112.84 (16)
C3A—C3—H3A	106.8	C1—C10B—C3A	105.65 (16)
C4—C3A—C14	112.35 (16)	C10A—C10B—H10B	105.6
C4—C3A—C10B	106.94 (16)	C1—C10B—H10B	105.6
C14—C3A—C10B	112.05 (16)	C3A—C10B—H10B	105.6
C4—C3A—C3	116.49 (17)	C12—C11—C13	109.3 (2)
C14—C3A—C3	108.49 (16)	C12—C11—C3	110.2 (2)
C10B—C3A—C3	99.89 (15)	C13—C11—C3	114.5 (2)
C3A—C4—C5	111.34 (17)	C12—C11—H11	107.5
C3A—C4—H4A	109.4	C13—C11—H11	107.5
C5—C4—H4A	109.4	C3—C11—H11	107.5
C3A—C4—H4B	109.4	C11—C12—H12A	109.5
C5—C4—H4B	109.4	C11—C12—H12B	109.5
H4A—C4—H4B	108	H12A—C12—H12B	109.5
C4—C5—C5A	115.89 (18)	C11—C12—H12C	109.5
C4—C5—H5A	108.3	H12A—C12—H12C	109.5
C5A—C5—H5A	108.3	H12B—C12—H12C	109.5
C4—C5—H5B	108.3	C11—C13—H13A	109.5
C5A—C5—H5B	108.3	C11—C13—H13B	109.5
H5A—C5—H5B	107.4	H13A—C13—H13B	109.5
C15—C5A—C5	108.27 (19)	C11—C13—H13C	109.5
C15—C5A—C6	109.69 (18)	H13A—C13—H13C	109.5
C5—C5A—C6	107.63 (18)	H13B—C13—H13C	109.5
C15—C5A—C10A	109.16 (19)	O2—C14—O1	121.61 (18)
C5—C5A—C10A	107.99 (17)	O2—C14—C3A	124.46 (18)
C6—C5A—C10A	113.94 (17)	O1—C14—C3A	113.90 (17)
C7—C6—C5A	120.54 (18)	C5A—C15—H15A	109.5
C7—C6—H6A	107.2	C5A—C15—H15B	109.5
C5A—C6—H6A	107.2	H15A—C15—H15B	109.5
C7—C6—H6B	107.2	C5A—C15—H15C	109.5
C5A—C6—H6B	107.2	H15A—C15—H15C	109.5
H6A—C6—H6B	106.8	H15B—C15—H15C	109.5
O3—C7—C6	108.13 (17)	C8—C16—H16A	109.5
O3—C7—C8	110.08 (17)	C8—C16—H16B	109.5
C6—C7—C8	116.42 (17)	H16A—C16—H16B	109.5
O3—C7—H7	107.3	C8—C16—H16C	109.5
C6—C7—H7	107.3	H16A—C16—H16C	109.5
C8—C7—H7	107.3	H16B—C16—H16C	109.5
			
C10B—C1—C2—C3	8.2 (2)	C9—C10—C10A—C10B	−79.8 (3)
C1—C2—C3—C11	149.26 (18)	C9—C10—C10A—C5A	47.8 (3)
C1—C2—C3—C3A	18.8 (2)	C15—C5A—C10A—C10	60.6 (2)
C11—C3—C3A—C4	81.3 (2)	C5—C5A—C10A—C10	178.06 (18)
C2—C3—C3A—C4	−152.25 (18)	C6—C5A—C10A—C10	−62.4 (2)
C11—C3—C3A—C14	−46.6 (2)	C15—C5A—C10A—C10B	−170.67 (17)
C2—C3—C3A—C14	79.8 (2)	C5—C5A—C10A—C10B	−53.2 (2)
C11—C3—C3A—C10B	−164.02 (18)	C6—C5A—C10A—C10B	66.3 (2)
C2—C3—C3A—C10B	−37.57 (19)	C10—C10A—C10B—C1	−43.1 (2)
C14—C3A—C4—C5	−68.2 (2)	C5A—C10A—C10B—C1	−172.95 (17)
C10B—C3A—C4—C5	55.2 (2)	C10—C10A—C10B—C3A	−169.03 (16)
C3—C3A—C4—C5	165.83 (16)	C5A—C10A—C10B—C3A	61.1 (2)
C3A—C4—C5—C5A	−55.1 (2)	C2—C1—C10B—C10A	−162.12 (17)
C4—C5—C5A—C15	170.12 (19)	C2—C1—C10B—C3A	−32.9 (2)
C4—C5—C5A—C6	−71.4 (2)	C4—C3A—C10B—C10A	−60.5 (2)
C4—C5—C5A—C10A	52.0 (2)	C14—C3A—C10B—C10A	63.1 (2)
C15—C5A—C6—C7	−56.7 (3)	C3—C3A—C10B—C10A	177.76 (15)
C5—C5A—C6—C7	−174.27 (18)	C4—C3A—C10B—C1	165.97 (16)
C10A—C5A—C6—C7	66.0 (2)	C14—C3A—C10B—C1	−70.5 (2)
C5A—C6—C7—O3	166.72 (16)	C3—C3A—C10B—C1	44.2 (2)
C5A—C6—C7—C8	−68.8 (2)	C2—C3—C11—C12	62.0 (3)
O3—C7—C8—O4	69.69 (19)	C3A—C3—C11—C12	−175.7 (2)
C6—C7—C8—O4	−53.8 (2)	C2—C3—C11—C13	−174.3 (2)
O3—C7—C8—C9	−170.74 (16)	C3A—C3—C11—C13	−52.1 (3)
C6—C7—C8—C9	65.8 (2)	C4—C3A—C14—O2	165.1 (2)
O3—C7—C8—C16	−48.1 (2)	C10B—C3A—C14—O2	44.7 (3)
C6—C7—C8—C16	−171.59 (18)	C3—C3A—C14—O2	−64.7 (3)
O4—C8—C9—C10	67.3 (3)	C4—C3A—C14—O1	−17.0 (2)
C16—C8—C9—C10	−174.3 (3)	C10B—C3A—C14—O1	−137.48 (19)
C7—C8—C9—C10	−50.8 (3)	C3—C3A—C14—O1	113.2 (2)
C8—C9—C10—C10A	2.5 (4)		

### Hydrogen-bond geometry (Å, °)

**Table:**

*D*—H···*A*	*D*—H	H···*A*	*D*···*A*	*D*—H···*A*
O1—H1···O4^i^	0.82	1.80	2.613 (3)	170
O3—H3···O2^ii^	0.82	2.01	2.825 (3)	173
O4—H4···O3^iii^	0.82	1.94	2.752 (3)	172
C1—H1B···O2	0.97	2.44	2.889 (4)	108
C5—H5B···O1	0.97	2.51	3.054 (4)	116
C6—H6B···O4	0.97	2.52	2.901 (3)	104
C16—H16A···O3	0.96	2.46	2.809 (4)	101

Symmetry codes: (i) −*x*+1/2, −*y*+2, *z*−1/2; (ii) −*x*+1/2, −*y*+2, *z*+1/2; (iii) *x*+1/2, −*y*+5/2, −*z*+2.
